# H3K9ac and HDAC2 Activity Are Involved in the Expression of Monocarboxylate Transporter 1 in Oligodendrocyte

**DOI:** 10.3389/fnmol.2017.00376

**Published:** 2017-11-14

**Authors:** Qingwei Lai, Wantong Du, Jian Wu, Xiao Wang, Xinyu Li, Xuebin Qu, Xiuxiang Wu, Fuxing Dong, Ruiqin Yao, Hongbin Fan

**Affiliations:** ^1^Xuzhou Key Laboratory of Neurobiology, Jiangsu Key Laboratory of New Drug Research and Clinical Pharmacy, Xuzhou Medical University, Xuzhou, China; ^2^Department of Neurology, Affiliated Hospital of Xuzhou Medical University, Xuzhou, China

**Keywords:** epigenetics, acetylation, H3K9, monocarboxylate transporter 1, oligodendrocyte

## Abstract

Recently, it is reported that monocarboxylate transporter 1 (MCT1) plays crucial role in oligodendrocyte differentiation and myelination. We found that MCT1 is strongly expressed in oligodendrocyte but weakly expressed in oligodendrocyte precursors (OPCs), and the underlying mechanisms remain elusive. Histone deacetylases (HDACs) activity is required for induction of oligodendrocyte differentiation and maturation. We asked whether HDACs are involved in the regulation of MCT1 expression. This work revealed that the acetylation level of histone H3K9 (H3K9ac) was much higher in *mct1* gene (*Slc16a1*) promoter in OPCs than that in oligodendrocyte. H3K9ac regulates MCT1 expression was confirmed by HDAC acetyltransferase inhibitors trichostatin A and curcumin. Of note, there was a negative correlation between H3K9ac and MCT1 expression in oligodendrocyte. Further, we found that the levels of HDAC1, 2, and 3 protein in oligodendrocyte were obviously higher than those in OPCs. However, specific knockdown of HDAC2 but not HDAC1 and HDAC3 significantly decreased the expression of MCT1 in oligodendrocyte. Conversely, overexpression of HDAC2 remarkably enhanced the expression of MCT1. The results imply that HDAC2 is involved in H3K9ac modification which regulates the expression of MCT1 during the development of oligodendrocyte.

## Introduction

Oligodendrocyte precursor cells (OPCs) derived from neuroepithelial cells of the early embryonic neural tube ventricular zone. With the development of central nervous system (CNS), OPCs gradually migrate, proliferate, and differentiate into mature oligodendrocyte with myelination capability. The failure of oligodendrocyte myelination or remyelination results in a diversity of developmental and acquired neurological diseases such as leukodystrophies, cerebral palsy, and multiple sclerosis (MS) (Trapp et al., 1998; Berger et al., 2001).

Monocarboxylate transporter 1 (MCT1, also referred to *Slc16a1*), along with MCT2, MCT4, are extracellular membrane protein which transport pyruvate, lactate, and ketone to produce ATP to match particular metabolic needs in the CNS (Pierre and Pellerin, 2005; Halestrap and Wilson, 2012). It was most recently reported that high expression of MCT1 was in mature oligodendrocyte, while it was scarcely expressed in astrocytes, nor was it found in NG2 positive immature oligodendrocyte (Rinholm et al., 2011). MCT1 not only plays essential roles in oligodendrocyte development and myelination, but also provides energy metabolites to neurons. Blocking MCT1-mediated lactate transport resulted in the loss of axonal function and neurons degeneration (Sanchez-Abarca et al., 2001; Rinholm et al., 2011; Lee et al., 2012). Although the roles of MCT1 has been confirmed in oligodendrocyte development and maintaining neuronal function, the underlying mechanisms controlling MCT1 expression in oligodendrocyte and OPCs remain unclear (Morrison et al., 2013; Saab et al., 2013; Klosinski et al., 2015).

Recent evidence indicated that epigenetic factors play a critical regulatory role for oligodendrocyte maturation (Emery and Lu, 2015; Hernandez et al., 2016; Zhang et al., 2016). Acetylation of nucleosomal histones modified by histone deacetylases (HDACs) and histone acetyltransferases (HATs) have identified a role for epigenetic mechanisms in the regulation of oligodendrocyte development. HATs/HDACs can be recruited to their binding sites to form multiprotein transcriptional activators or repressor complexes to modulate gene expression (Gregoretti et al., 2004). Remarkably little is known about the function of HATs in oligodendrocyte development, however, much of the research has already been implicated that HDACs activity is indispensable for oligodendrocyte differentiation and myelin-related gene expression (Marin-Husstege et al., 2002; Balasubramanyam et al., 2004; Shen et al., 2005). Whether acetylation of *Slc16a1* promoter controls the expression of MCT1 in OPCs and oligodendrocyte, and which types of HDACs are involved in the modification remain elusive.

To clarify the potential epigenetic regulation mechanisms in MCT1 expression in oligodendrocyte, we performed chromatin immunoprecipitation-polymerase chain reaction (ChIP-PCR) to determine acetylation levels of *Slc16a1* promoter regions. The relationship of acetyl-histone H3 Lys9 (H3K9ac) and MCT1 expression was confirmed by curcumin, a histone acetyltransferase inhibitor (Balasubramanyam et al., 2004), and trichostatin A (TSA), a deacetylase inhibitor (Zhang et al., 2004). Further, it was defined that HDAC2 is involved in H3K9ac modification of *Slc16a1* promoter which is responsible for MCT1 expression. Thus, this work provides a new insight into how histone acetylation modification takes part in the expression of MCT1 in oligodendrocyte.

## Materials and Methods

### Ethics Statement

The animals in this study followed guidelines and regulations set by the National Institutes of Health guide (NIH) for the Care and Use of Laboratory Animals. Animal experiments were approved by the Institutional Animal Care and Use Committee of Xuzhou Medical University (No. 201607). We made all efforts to minimize animal suffering and reduce the number of animals used.

### Cell Culture

As we have previously described for OPCs proliferation culture (Wang et al., 2011). Fresh cerebral cortex samples were isolated from P1-2 Sprague-Dawley rat pup and placed into ice-cold DMEM/F12 medium (1:1, GIBCO) containing penicillin (50 μg/ml) and streptomycin (50 μg/ml). Single cell suspension was prepared using 70-μm cell strainer. After centrifugation (1,000 rpm for 10 min), cells were then resuspended in DMEM/F12 medium supplemented with 10% fetal bovine serum (FBS), incubated at 37°C in a humid atmosphere of 5% CO_2_ and 95% air for 8 days. The same medium was changed every 2–3 days. Eight days later, OPCs were purified by shaking the flasks on a horizontal orbital shaker. In order to remove adherent microglial cells and macrophages, the flasks were shaken for 1 h at 37°C and the medium was replaced with fresh DMEM/F12 medium containing 10% FBS. After shaking vigorously for 16–18 h at 220 rpm, the cell suspension was transferred to an untreated Petri dish, then incubation for 0.5 h to allow microglia and astrocytes to adhere to walls of Petri dish. OPCs floating in the medium was transferred to a tube and spun down at 1,000 rpm for 10 min. Cells were resuspended in DMEM/F12 medium supplemented with 2% B27, 10 ng/ml platelet derived growth factor AA (PDGF-AA, GIBCO), and 10 ng/ml basic fibroblast growth factor (bFGF, GIBCO) and then plated at 10,000 cells/cm^2^ on poly-L-lysine coated 25 cm^2^ flasks, and the medium was changed every 2 days. For oligodendrocyte differentiation, bFGF and PDGF-AA were removed from the OPCs medium and replaced by 10% FBS. The medium was changed every second day.

### Curcumin and TSA Treatment

Oligodendrocyte precursors were cultured for 48 h in the presence of bFGF and PDGF-AA and then induced to differentiate for 24 h by removing the mitogen from the medium with or without TSA (50 and 100 nM, Sigma-Aldrich) or curcumin (50 and 100 μM, Sigma-Aldrich). The same volume of dimethyl sulfoxide (DMSO, 50 μl, Sigma-Aldrich) was added to the control group. The effect of TSA or curcumin on H3K9ac of *Slc16a1* promoter was detected by ChIP-PCR. At the same time, MCT1 expression was examined by real-time reverse transcriptase (rtRT)-PCR, western blot, and immunocytochemistry.

### RNA Interference Transfection

For transient transfection, small interfering RNA (siRNA) specific for HDAC1siRNA (cat. no. sc-270070), HDAC2siRNA (cat. no. sc-270150), HDAC3siRNA (cat. no. sc-270161), and control siRNA (cat. no. sc-37007) were purchased from Santa Cruz Biotechnology, Inc. Transfections were performed using Lipofectamine RNAiMax (Invitrogen; Thermo Fisher Scientific, Inc.) according to the instruction of manufacturer. In brief, 1 × 10^6^ cells were grown in a culture dish at a density of 60–80% confluence and were transfected with 0.5 nmol siRNA mixed with 15 μl Geneporter 2 Transfection Reagent (GTS, San Diego, CA, United States). After 6 h of transfection, the cells were cultured for another 18 h and harvested for protein expression analyses. Control siRNA was available as negative control for evaluating RNAi off-target effects, it consists of a scrambled sequence that will not cause the specific degradation of any known cellular mRNA.

### Generation and Transfection of Recombinant Plasmid

Target gene HDAC2 was detected by RT-PCR. Primers designed and utilized for HDAC2 were as follows: Forward sequence: 5′-GGGAATTCATGGCGTACAGTCAAGGAGG-3′ and Reverse sequence: 5′-GGGGTACCGGGAGTCAAATTCAAGGGTTGT-3′. Agarose gel electrophoresis was performed to determine the amplified PCR products, and the bands were visualized under UV light. To produce pEGFP-C2-HDAC2 recombinant plasmid, EcoR 1 and Kpn 1 restriction enzymes were used to cut the purified PCR segment, then the segment was subcloned into the vector pEGFP-C2. HEK 293T cells were transfected with pEGFP-C2-HDAC2 plasmid, and the correct fragment produced by Kpn I/EcoR I co-digestion was confirmed by sequence analysis. Oligodendrocyte transfected with empty vector plasmid was considered as control group.

### Immunocytochemistry

Cells were fixed in 4% paraformaldehyde for 15 min at room temperature. After washing three times with 0.01 mmol/l phosphate buffer saline (PBS), the cells were blocked with PBS containing 5% donkey serum and 0.2% Triton X-100 for 1 h and then incubated overnight at 4°C with mouse anti-A2B5 (1:200, Chemicon, cat. no. MAB312R), mouse anti-MBP (1:200, Abcam, cat. no. ab62631), and goat anti-MCT1 (1:100, Santa Cruz Biotechnology, SC-14917) primary antibodies. Negative control was treated with an equivalent volume of diluent solution without primary antibody. After washing with PBS, cells were incubated for 1 h at 4°C with secondary antibodies CY2-conjugated donkey anti-goat IgG and CY3-conjugated goat anti-mouse IgG (all 1:200; all from Amersham Biosciences Ltd., GE Healthcare, United States). Nuclei were stained with 4,6-diamidino-2-phenylindole, DAPI (Sigma, United States). Fluorescence images were captured with a Zeiss Axioskop 40 microscope (Carl Zeiss, Oberkochen, Germany). The expression of MCT1 was evaluated according to immunofluorescence staining result, integral optical density (IOD) and region of interest were measured by Image-Pro Plus 6.0 software (Media Cybernetics Inc., United States). Values of optical density in individual cells represented the quantity of MCT1 protein, and were calculated by the equation: ΣIOD/ΣArea. In this study, ΣIOD is the sum of the IOD of A2B5/MCT1 or MBP/MCT1 double-positive cells in the photograph and ΣArea is the total area of A2B5- or MBP-positive cells in the photograph.

### Western Blot Analysis

Cells were rinsed with ice-cold PBS, collected, and spun down at 1,000 rpm for 10 min at 4°C, then resuspended in ice-cold lysis buffer containing 0.1% Nonidet P-40, 1 mM dithiothreitol, 10 mM EDTA, 40 mM Tris–HCl (pH 7.4), 120 mM NaCl, and protease inhibitors for 30 min on ice. Cell lysates were centrifuged at 15,000 rpm for 5 min at 4°C and the protein content in the supernatant was determined by a BIO-RAD Protein Assay Kit. Equal amounts of protein (20 μg) were separated by 10 or 12.5% SDS-PAGE gels and transferred onto nitrocellulose membranes. The membranes were first blocked with 5% non-fat dry milk for 1 h and then incubated overnight at 4°C with the following primary antibodies against MCT1 (1:500, Santa Cruz Biotechnology, SC-14917), HDAC1 (1:1000, Cell Signaling Technology, cat. no. 34589), HDAC2 (1:200, Cell Signaling Technology, cat. no. 57156), HDAC3 (1:3000, Abcam, cat. no. ab32369), HDAC8 (1:3000, Abcam, cat. no. ab187139), and β-actin (1:1000, Arigo, cat. no. ARG62346). After washing with Tris–Tween-buffered saline, the membrane was then incubated with fluorescent secondary antibodies for 1.5 h. The blots were visualized by Odyssey Infrared Imaging System (LI-CON) and the density of protein bands was measured by Image J. The expression levels of HDACs and β-actin protein were quantified by densitometry and each HDAC band was normalized against the corresponding level of β-actin.

### DNA Extraction and RNA Extraction

Genomic DNA and RNA were extracted from OPCs and oligodendrocyte with QIAamp DNA Mini Kit (Qiagen, Valencia, CA, United States) and Trizol reagent (Invitrogen, Carlsbad, CA, United States) according to the manufacturer’s instructions, respectively.

### Real-Time Reverse Transcriptase Polymerase Chain Reaction (rtRT-PCR)

The quantitative one-step RT-PCR Kit (Vazyme Biotech Co., Ltd., China) and related primers (synthesized by Sangon Biotech, China) were used for detecting the *Slc16a1* expression according to a standardized protocol as previous described in Roche Applied Science LightCycle^TM^480 (Roche Science, Switzerland). The sense and antisense primers for MCT1 were: Forward sequence: 5′-AGGTCCTATCAGCAGTATCT-3′ and Reverse sequence: 5′-AGTTCCTGCACCGTGTTACA-3′. The expression level of ribosomal RNA18s (18sRNA) was acted as a reference for samples. The sense and antisense primers for18sRNA used as an internal control were: Forward sequence: 5′-CCTGGATACCGCAGCTAGGA-3′ and Reverse sequence: 5′-GCGGCGCAATACGAATGCCCC-3′. The PCR cycling conditions were 35 cycles of denaturation at 95°C for 3 s, annealing at 57°C for 30 s, extension at 72°C for 30 s, and followed by a final extension at 72°C for 8 min. Relative amount of MCT-1 mRNA expression was calculated according to the standard 2^-ΔΔC_t_^ method by normalization to the 18sRNA mRNA level.

### Chromatin Immunoprecipitation- Polymerase Chain Reaction (ChIP-PCR)

With the instructions of EZ-ChIP Chromatin Immunoprecipitation Kit (Millipore), ChIP was performed with rabbit anti-H3K9ac polyclonal antibody (1:1000, Millipore, cat. no. 07-352) and the DNA precipitated with the target antibody was detected by conventional PCR and RT-PCR. According to our previously reports (Yu et al., 2014), PCR was conducted in a final volume of 25 μl containing 12.5 μl rTaq or SYBR Premix Ex Taq^TM^ II (Takara, Otsu, Japan), 1 μl each of forward and reverse primers (10 μM), and 3 μl DNA template under the following conditions: denaturation at 95°C for 2 min and then subjected to 30 or 40 cycles of amplification (95°C for 15 s, 60°C for 30 s, 72°C for 20 s). After that, the PCR products were detected by agarose gel electrophoresis. Relative data quantification was performed using the 2^-ΔΔC_t_^ method, the data were calculated by the formula: % Input = 2 ^(CtInput-CtChIP)^ × input dilution factor × 100 and expressed in the form of % input. The sequences of PCR primers used for *Slc16a1* were list in **Table 1**.

**Table 1:** ChIP-PCR Primer sequences.

Gene name	Primer sequence	Annealing temperature (°C)	Primer length	Position (bp)
Slc16a1-p1	F: 5′-GGTATGCAGTAATCGCCTTGG-3′ R: 5′-CGCACCATCGGTGTCAATAT-3′	60	237	-1469 ~-1449 -1252 ~-1223
Slc16a1-p2	F: 5′-CCACAGTGGCTGATGTAGCG-3′ R: 5′-TCGAGGGACTGGACAGCATT-3′	60	131	-537 ~-518 -426 ~-407
Slc16a1-p3	F: 5′-CATGAAACTGTCCTAATCCCACT-3′ R: 5′-TACGCAAGTATCTCGTCACAGAG-3′	60	211	-776 ~-753 -588 ~-566

### Statistical Analysis

All experiments were performed with SPSS 16.0 software and the data are expressed as mean ± SEM. Statistical analyses were conducted with two-tailed *T*-tests for comparing two sample means, one-way analysis of variance (ANOVA) followed by Tukey’s HSD post-test for comparison of multiple sample means, and bivariate correlation analysis for the correlation of two factors. Each representative result is from three independent experiments. *P* < 0.05 was considered to indicate statistical significance for all tests.

## Results

### MCT1 Was Strongly Expressed in Mature Oligodendrocyte, But Weakly in OPCs

**FIGURE 1:**
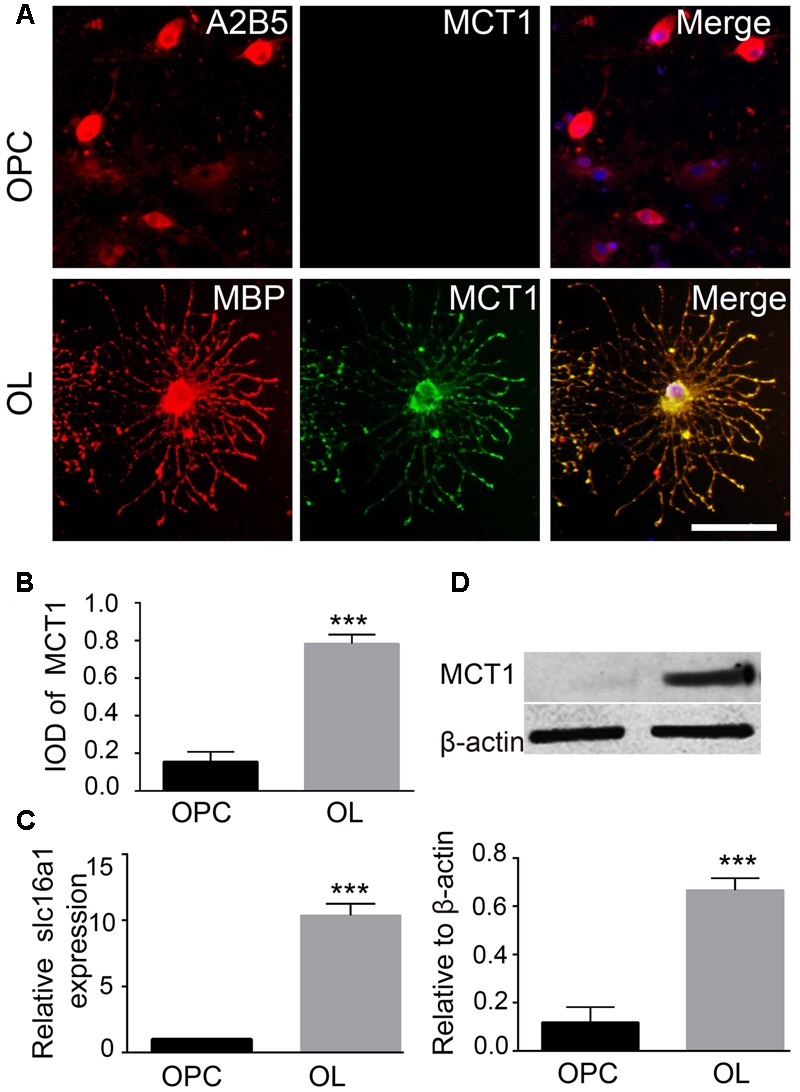
Detection of MCT1 expression in OPCs and mature oligodendrocyte. **(A)** Immunocytofluorescent staining showed the expression of MCT1 in A2B5 positive OPCs and MBP positive oligodendrocyte. Scale: 20 μm. **(B)** The IOD MCT1^+^/A2B5^+^ and MCT1^+^/MBP^+^ area was assessed by counting five random fields (in duplicate wells) per group (^∗∗∗^*P* < 0.001). **(C)** RT-PCR analysis showed that the MCT1 mRNA expression was higher in oligodendrocyte than that in OPCs (^∗∗∗^*P* < 0.001). **(D)** The level of MCT1 protein in OPCs and oligodendrocyte was examined by western blot, and the quantitative analysis showed that MCT1 protein in oligodendrocyte was also significantly higher than that in OPCs (^∗∗^*P* < 0.001). Representative data from three independent experiments (*N* = 4 for per group in each experiment).

The expression of MCT1 in OPCs and mature oligodendrocyte was detected by immunofluorescence, RT-PCR, and western blot. Double immunostaining for MCT1/A2B5 (OPCs marker) and MCT1/MBP (mature oligodendrocyte marker) showed that few of A2B5-positive cells were co-labeled with MCT1, but most of the MBP-positive cells co-expressed MCT1 (**Figure 1A**). The IOD of MCT1^+^/MBP^+^ in oligodendrocyte was significantly higher than the IOD of MCT1^+^/A2B5^+^ in OPCs (**Figure 1B**). Meanwhile, the levels of MCT1 mRNA and protein in oligodendrocyte were also remarkably higher than those in OPCs (**Figures 1C,D**). The results indicated that MCT1 was strongly expressed in mature oligodendrocyte but weakly in OPCs.

### The Level of H3K9ac of Slc16a1 Promoter in OPCs and Oligodendrocyte

**FIGURE 2:**
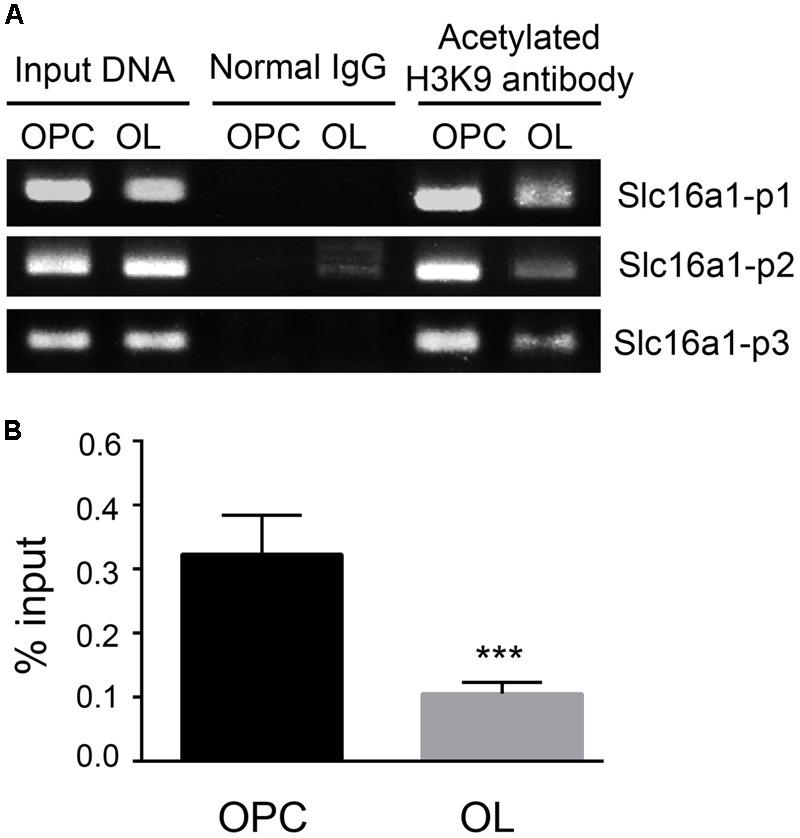
The level of H3K9ac in the *Slc16a1* promoter in OPCs and oligodendrocyte. **(A)** After chromatins in OPCs and oligodendrocyte were cross-linked and broken, ChIP analyses were carried out with H3K9 antibody or normal rabbit IgG (negative control). After that, the immunoprecipitated DNAs and the input DNA were assayed using conventional PCR with specific primers (*Slc16a1* P1, P2, and P3). The PCR products were separated on 2% agarose gel electrophoresis. **(B)** After RT-PCR, the ChIP DNA was normalized to the input DNA, and the data are expressed as the percentage of input chromatin. Compared to oligodendrocyte, H3K9ac of the *Slc16a1* promoter in OPCs was significantly higher (^∗∗∗^*P* < 0.001). Representative data from three independent experiments (*N* = 3 for per group in each experiment). All the results from three primers were pooled to quantify the input DNA.

The acetylation status of H3K9 in the *Slc16a1*promoter region in OPCs and oligodendrocyte was detected by ChIP-PCR (**Figure 2A**). The acetylation level of H3K9 in the *Slc16a1*promoter in oligodendrocyte was obviously lower than that in OPCs (*P* < 0.001) (**Figure 2B**), indicating that the activity of *Slc16a1* promoter might be regulated by H3K9ac.

### TSA and Curcumin Altered H3K9ac of the Slc16a1 Promoter and MCT1 Expression

**FIGURE 3:**
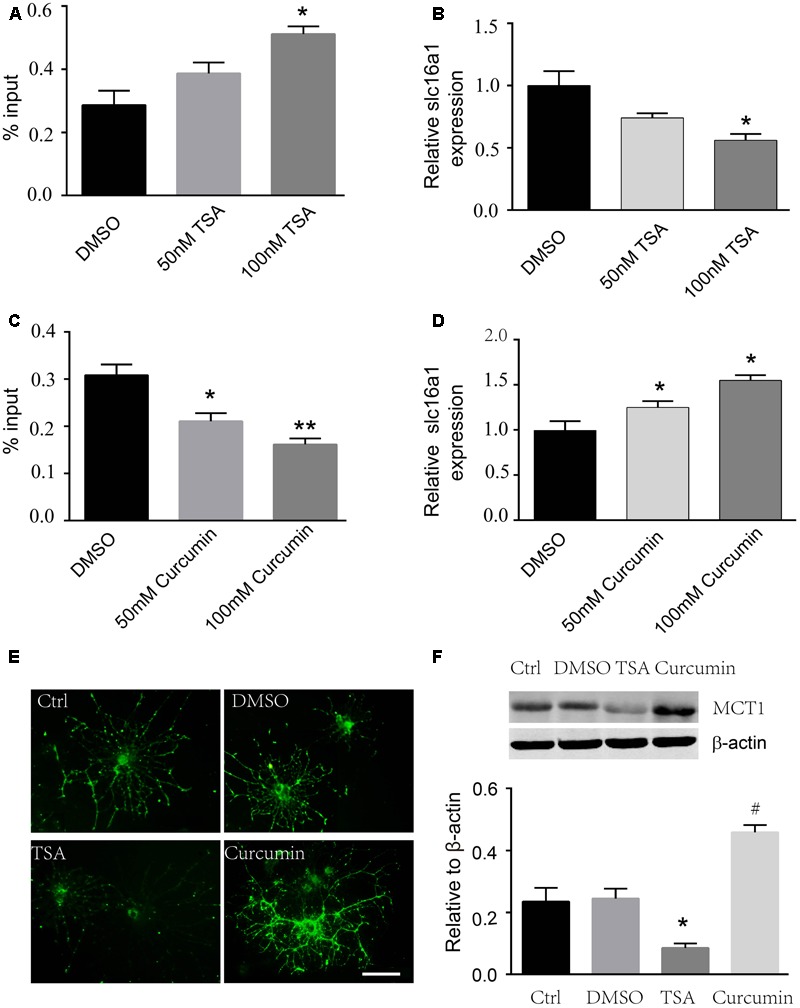
Curcumin and TSA altered H3K9ac in the *Slc16a1* promoter and MCT1 expression in oligodendrocyte. **(A)** ChIP-PCR showed that H3K9ac in the *Slc16a1* promoter increased with 100 nM TSA treatment (^∗^*P* < 0.05). **(B)** *Slc16a1* mRNA relative expression in oligodendrocyte was markedly decreased in a dose-dependent manner with TSA treatment compared to the control group (^∗^*P* < 0.05, 100 nM TSA vs. Ctrl). **(C)** H3K9ac in the *Slc16a1* promoter in oligodendrocyte decreased significantly when treated with curcumin (^∗^*P* < 0.05, 50 μM curcumin vs. Ctrl; ^∗∗^*P* < 0.01, 100 μM curcumin vs. Ctrl). **(D)** *Slc16a1* mRNA relative expression showed an increase with 50 μM and 100 μM curcumin treatment, respectively (^∗^*P* < 0.05; ^∗^*P* < 0.05). **(E)** Representative images for MCT1 immunocytofluorescent staining in normal control, vehicle control, 100 nm TSA, and 100 μm curcumin-treated oligodendrocyte. Scale: 10 μm. **(F)** Western blot bands and quantitative analysis showed that MCT1 protein was significantly decreased in TSA group (^∗^*P* < 0.05) and increased in curcumin group (^#^*P* < 0.05). Representative data from three independent experiments (*N* = 3 for per group in each experiment).

To prove whether H3K9ac is associated with the regulation of the activity of the *Slc16a1* promoter, we used curcumin and TSA to evaluate the effect of acetylation status of the *Slc16a1* promoter on MCT1 expression in immature oligodendrocyte (cultured 2 days in differentiation medium). ChIP-PCR validated that TSA markedly increased the acetylation level of H3K9 in the *Slc16a1* promoter in a dose-dependent manner. H3K9ac level was the highest in the 100 nM TSA group (**Figure 3A**). Meanwhile, RT-PCR results revealed that TSA downregulated *Slc16a1* mRNA expression (**Figure 3B**). The correlation analysis revealed that H3K9ac in the *Slc16a1* promoter has significant negative correlation with *Slc16a1* mRNA expression (*r* = 0.895, *P* < 0.01). Fifty and hundred micrometer curcumin treatment significantly decreased H3K9ac and increased *Slc16a1* mRNA expression (**Figures 3C,D**). The correlation analysis indicated a significant negative correlation between H3K9ac in the *Slc16a1* promoter and *Slc16a1* mRNA expression (*r* = 0.744, *P* < 0.05). The function of TSA and curcumin-regulating MCT1 expression was further confirmed by immunostaining and western blot (**Figures 3E,F**). TSA significantly decreased the expression of MCT1 and curcumin increased the expression of MCT1 compared with the DMSO group (*P* < 0.05 TSA vs. DMSO, *P* < 0.05 curcumin vs. DMSO).

### HDAC2 Is Involved in H3K9ac Modification in the *Slc16a1* Promoter

**FIGURE 4:**
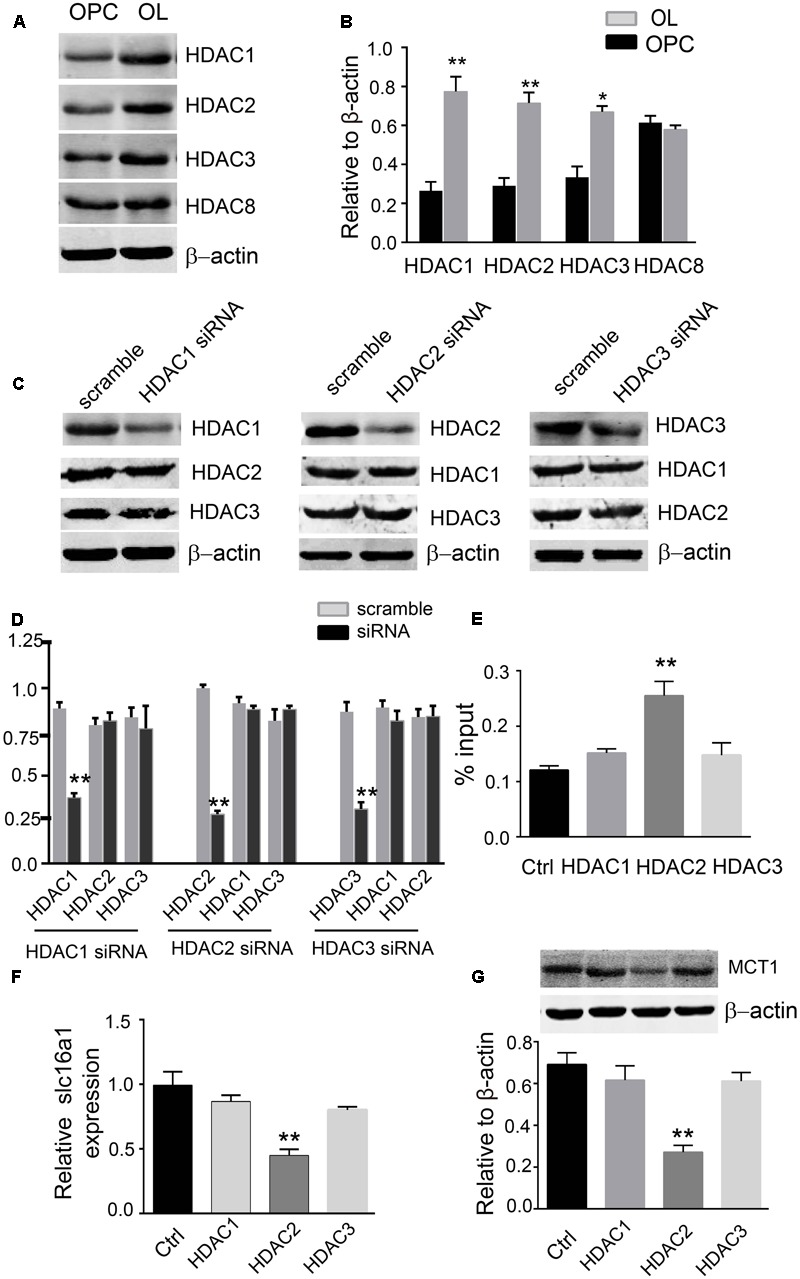
HDAC2 regulates H3K9ac in the *Slc16a1* promoter and MCT1 expression. **(A)** Representative western blot bands for HDAC1, 2, 3, and 8 proteins in OPCs and oligodendrocyte. **(B)** The levels of HDAC1, 2, and 3 protein were obviously higher in oligodendrocyte than those in OPCs (^∗^*P* < 0.05; ^∗∗^*P* < 0.01). No evident change was observed for HDAC8 protein between OPCs and oligodendrocyte. **(C,D)** Western blot was performed to confirm the commercial siRNAs are specific to the HDAC that they target (^∗∗^*P* < 0.01). **(E)** Knockdown of HDAC2 significantly increased H3K9ac in the *Slc16a1* promoter compared to the control group (^∗^*P* < 0.05). **(F,G)** Both *Slc16a1* mRNA and MCT1 protein were dramatically decreased in HDAC2 siRNA-treated group compared to the scramble group (^∗∗^*P* < 0.01; ^∗∗^*P* < 0.01). Representative data from three independent experiments (*N* = 4 for per group in each experiment).

In order to clarify which types of HDACs might be involved in H3K9ac modification, we focused on class I HDACs and detected the expression differences of HDAC1, 2, 3, and 8 in OPCs and oligodendrocyte. Western blot analysis demonstrated that the HDAC1, 2, and 3 proteins were clearly higher than those in OPCs, but the levels of HDAC8 were not altered between OPCs and oligodendrocyte (**Figures 4A,B**). The expression of HDAC1, 2, and 3 proteins in oligodendrocyte was effectively downregulated using their siRNAs. The siRNAs used in this study are specific to the HDAC that they target due to each siRNA did not alter the expression of the other related HDACs that were not directly targeted by that siRNA (**Figures 4C,D**). Furthermore, downregulation of HDAC2 expression evidently increased the H3K9ac in the *Slc16a1* promoter (**Figure 4E**) and decreased the expression of MCT1 mRNA and protein. But HDAC1 and HDAC3 knockdown did not change the levels of H3K9ac and the expression of MCT1 (**Figures 4F,G**).

### HDAC2 Overexpression Promoted MCT1 Expression

**FIGURE 5:**
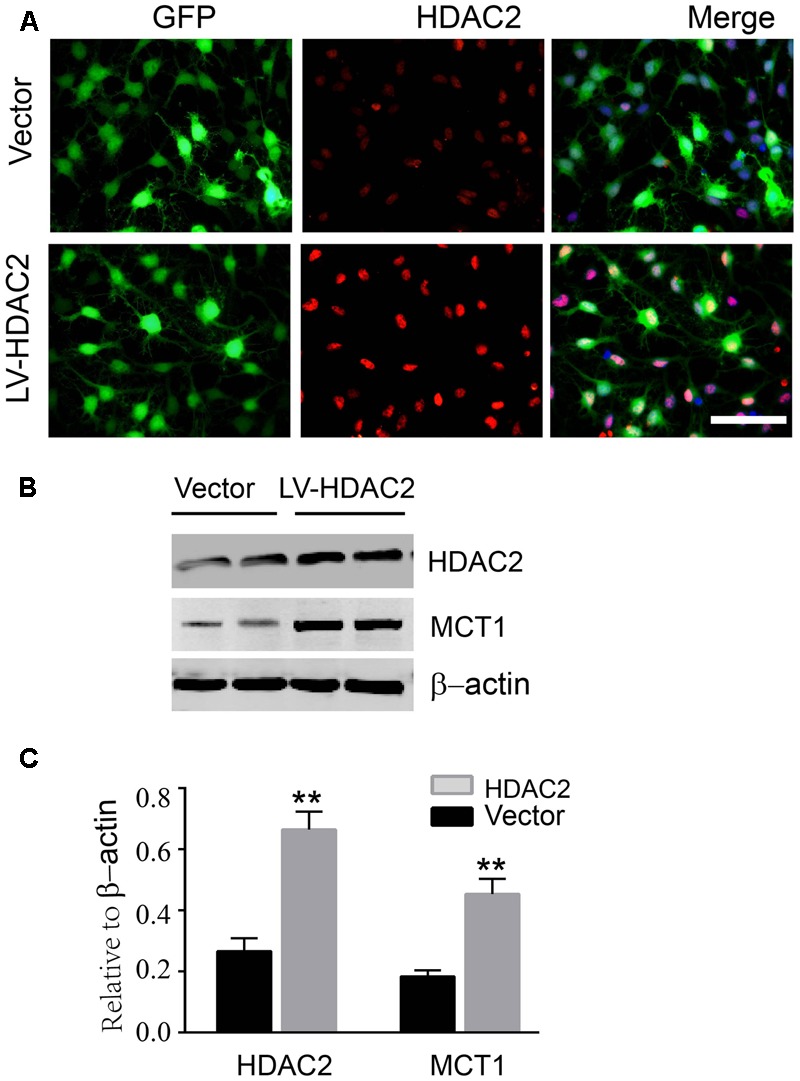
Overexpression of HDAC2 increased the expression of MCT1. **(A)** Representative immunofluorescent staining for HDAC2 in empty vector plasmid and HDAC2 constructed plasmid transfected immature oligodendrocyte. Scale: 20 μm. **(B,C)** HDAC2 constructed plasmid transfection obviously enhanced the level of HDAC2 compared to the vector group (^∗∗^*P* < 0.01). The level of MCT1 was also significantly increased by HDAC2 overexpression (^∗∗^*P* < 0.01). Representative data from three independent experiments (*N* = 4 for per group in each experiment).

To further confirm that HDAC2 is involved in the H3K9ac modification in the *Slc16a1* promoter and MCT1 expression, we treated the immature oligodendrocyte with HDAC2 recombinant plasmid. Immunostaining and western blot data showed that HDAC2 recombinant plasmid transfection markedly increased the expression of HDAC2 (**Figures 5A,C**). Meanwhile, the expression of MCT1 protein was also significantly upregulated by HDAC2 overexpression compared with the vector group (**Figures 5B,C**).

## Discussion

Remarkably, little is known about the mechanisms controlling MCT1 expression in oligodendrocyte. For the first time, we reported that H3K9ac levels at the *Slc16a1* promoter are higher in OPCs, where MCT1 expression is lower. H3K9ac level and MCT1 expression in oligodendrocyte are opposite to those in OPCs. We next show that pharmacological manipulations of HAT/HDACs alter H3K9ac levels and MCT1 expression. Based on the effects of HDAC siRNA manipulations on H3K9ac and MCT1 expression, we concluded that HDAC2 is involved in H3K9ac which regulates the expression of MCT1 in oligodendrocyte.

It has been reported that MCT1 was expressed in endothelial cells and glial fibrillary acidic protein (GFAP) – or S100β-positive astrocytes in the CNS (Pierre et al., 2000; Debernardi et al., 2003). However, it was recently reported that MCT1 was expressed predominately in oligodendrocyte, whereas virtually no expression of MCT1 was observed in adult CNS astrocytes, nor was it found in NG2-positive immature oligodendrocyte (Lee et al., 2012). In this study, it was affirmed that the mRNA and protein of MCT1 were strongly expressed in mature oligodendrocyte but weakly in OPCs. However, how the expression of MCT1 is regulated in oligodendrocyte remains elusive (Morrison et al., 2013; Saab et al., 2013; Klosinski et al., 2015).

Chromatin reorganization is an event crucial for the transition from progenitor to mature oligodendrocyte (Yu et al., 2013). The increasing evidence proved that histone modifications play a crucial role in oligodendrocyte development, myelination, and remyelination (Shen et al., 2008; Emery and Lu, 2015; Simons and Nave, 2016). Histone acetylation on lysine residues modified by HATs and HDACs is one of the best-characterized histone modifications. And a diversity of studies indicated that HDACs or their activities are required for oligodendrocyte differentiation and myelination processes (Marin-Husstege et al., 2002; Conway et al., 2012). Inhibiting HDACs activity with TSA and valproic acid can block oligodendrocyte differentiation both *in vitro* and *in vivo* (Marin-Husstege et al., 2002; Shen et al., 2005). The existence of HDACs on the proximal promoter region of the myelin protein gene in OPCs is a vital regulator of oligodendrocyte differentiation (Yu et al., 2010). Accordingly, we presumed that histone acetylation in the *Slc16a1* promoter might be involved in the expression of MCT1. ChIP-PCR confirmed that H3K9ac level in the *Slc16a1* promoter was obviously lower in oligodendrocyte than that in OPCs. And there was a significant negative correlation between H3K9ac in the *Slc16a1* promoter and MCT1 expression. But it is not clear which types of HDACs are required for H3K9ac in the *Slc16a1* promoter in oligodendrocyte.

Histone deacetylases are divided into four classes: ubiquitously expressed Class I (HDAC1, 2, 3, and 8), tissue-specific Class II (HDAC4, 5, 6, 7, 9, and 10), NAD-dependent Class III, and Class IV (HDAC11) (Grozinger and Schreiber, 2002; Gregoretti et al., 2004). It is widely stated in the literature that Class I HDACs are located in the nucleus and are ubiquitously expressed. However, each member of Class II HDACs shows at least some cytoplasmic localization, suggesting a major cytoplasmic functional role for Class II HDACs (Haberland et al., 2009; Schuetze et al., 2014). Our study found that H3K9ac is involved in the regulation of the activity of the *Slc16a1* promoter and *Slc16a1* transcription, we speculated that the HDACs are located in the nucleus regulates the H3K9ac level and *Slc16a1* transcription. In addition, it was also manifested that Class I HDACs are involved in neural stem/progenitor cell development (Jiang and Hesieh, 2014; Norwood et al., 2014). Consequently, in this study, we focused on Class I HDAC and investigated the expression of Class I HDAC in OPCs and oligodendrocyte. The results demonstrated there was a significant difference in HDAC1, 2, and 3 protein levels between OPCs and oligodendrocyte. But the levels of HDAC8 were not altered between OPCs and oligodendrocyte. We speculated that HDAC1, 2, and 3 might be involved in *Slc16a1* promoter histone acetylation. Using siRNA technology to specifically knockdown the expression of HDAC1, 2, and 3, it is HDAC2 but not HDAC1 and HDAC3 knockdown significantly increased the H3K9ac in the *Slc16a1* promoter and decreased the MCT1 expression. To further define that HDAC2 is involved in H3K9ac in the *Slc16a1* promoter and MCT1 expression, we treated the OPCs with a HDAC2 recombinant plasmid. The data showed that HDAC2 overexpression decreased the H3K9ac in the *Slc16a1* promoter and upregulated MCT1 expression, indicating that HDAC2 selectively regulates H3K9ac in the *Slc16a1* promoter in oligodendrocyte.

Yang et al. (2002) reported that HDAC1 and HDAC2 only have nuclear localization signal (NLS), while HDAC3 has both NLS and a nuclear export signal (NES), reflecting that HDAC3 might localize to the cytoplasm. Both RNAi-mediated knockdown study *in vitro* and HDAC1/2 knockout research *in vivo* revealed that HDAC1 and HDAC2 are critical for OPCs differentiation and myelination (Shen et al., 2008; Ye et al., 2009). HDAC1 can recruit Yin Yang1 (YY1) to the promoter of differentiation inhibitors such as Id2, Id4, and Hes5 to repress their transcription, which in turn facilitated oligodendrocyte differentiation (Shen et al., 2005; He et al., 2007). HDAC1 and HDAC2 prevent expression of Wnt signaling target genes Id2 and Id4 by disrupting the β-catenin/TCF transcription complex, to promote oligodendrocyte differentiation (Ye et al., 2009). HDAC3 is expressed in oligodendrocyte and its essential role in controlling oligodendrocyte and astrocyte lineages commitment was confirmed. HDAC3 cooperates with p300 to activate Olig2 to prime and maintain oligodendrocyte identity (Shen et al., 2005; Zhang et al., 2016). So far, there is no evidence indicates that HDAC8 is involved in oligodendrocyte development.

In the present study we found that HDAC2 is involved in H3K9ac and H3K9ac seems to be associated with MCT1 repression in oligodendrocyte, which is the opposite of how histone acetylation is known to regulate gene expression. Furthermore, while HAT and HDAC inhibitors do alter the expression of MCT1, it is very possible that this is an indirect effect and that something else that is altered by HAT/HDAC inhibition is actually responsible for transcriptional control of *Slc16a1*, rather than H3K9ac directly regulating that gene. For example, some proteins can be acetylated, such as p65, so HAT/HDAC inhibition could be altering the activity of other transcription factors that regulate *Slc16a1*. It is also likely that changes in histone acetylation of the promoter region of another transcription factor might in turn regulate transcription of *Slc16a1*.

## Conclusion

We found that downregulation of H3K9ac by HDAC2 is associated with MCT1 expression in oligodendrocyte. However, contrary to has been reported H3K9ac as a transcriptional activator across all different cell types and model systems, H3K9ac consistently correlates with transcriptional repression. Based on the current data we confer that histone acetylation indirectly regulates repression of MCT1 in OPCs. A better understanding of the molecular events how H3K9ac regulates MCT1 expression in oligodendrocyte remains to be defined. Taken together, this study will help to elucidate the mechanisms of MCT1 expression and oligodendrocyte development.

## Author Contributions

QL, WD, JW, and XW performed the experiments, collected and analyzed the data, and wrote the manuscript. XL, XQ, XW, and FD analyzed and interpreted the data and wrote the manuscript. HF and RY proposed the hypothesis, designed the experiments, significantly edited the manuscript, and provided the final approval of the manuscript. All authors read and approved the final version of the manuscript.

## Conflict of Interest Statement

The authors declare that the research was conducted in the absence of any commercial or financial relationships that could be construed as a potential conflict of interest.
